# Methodology for measuring behavior change dictates the perceived effectiveness of school-based WASH interventions

**DOI:** 10.3389/fpubh.2026.1833899

**Published:** 2026-08-31

**Authors:** Gracie Hornsby, Canon Pham, Jennifer Davis, Gary L. Darmstadt

**Affiliations:** 1 Department of Civil and Environmental Engineering, Stanford University, Stanford, CA, United States; 2 Woods Institute for the Environment, Stanford University, Stanford, CA, United States; 3 Department of Pediatrics, Stanford University School of Medicine, Stanford, CA, United States

**Keywords:** behavior change, observation, reporting bias, WASH, WASH in schools

## Abstract

**Introduction:**

Investments in the provision of water, sanitation, and hygiene (WASH) services in schools (WinS) can only generate health benefits if students use WASH facilities correctly and consistently. Valid and reliable measurement of student WASH behavior is thus critical for evaluating WinS interventions.

**Methods:**

This study examines the relative validity of direct observation versus reported (survey-based) measures of WASH behaviors in schools, using both primary data and a review of literature. We conducted a sub-analysis of a four-arm, cluster-randomized controlled trial among 200 primary schools in Uttar Pradesh, India, that tested the effect of WASH education and infrastructure operation and maintenance (O&M) on student handwashing and toilet use. We compared unannounced observations of student behavior against self-, peer-, and teacher-reports. We also stress-tested reported data against direct observation of WASH infrastructure and consumables availability. Finally, we conducted a comparative analysis of 42 published WinS studies to contextualize our primary findings.

**Results:**

Reported data indicated significant behavioral change and suggested that combining education with O&M created a synergistic effect. In contrast, direct observation revealed no significant changes in behavior under any treatment. Stress-testing showed that students frequently reported toilet use when toilets were observed to be locked, and soap use when soap was not observed to be present. Findings from our comparative analysis of WinS literature mirrors the findings of our primary data, revealing that 83% of intervention effects measured with reported data yielded positive results, compared to 58% when measured through observation.

**Discussion:**

We find that reported behavioral data systematically overstate the effectiveness of WinS interventions. Current WinS policy and practice is thus grounded in an evidence base that varies significantly depending on the measurement tools employed. Direct observation is less prone to reporting bias but is resource-intensive. Future research should prioritize objective measures of behavior, such as sensors or unannounced checks, to build more robust evidence for WinS policy and practice.

## 1 Introduction

The provision of water, sanitation, and hygiene (WASH) services in schools (WinS) is a critical component of global public health and development. Despite substantial domestic and international investment, a large infrastructure deficit remains. In 2023, a substantial proportion of schools worldwide still lacked access to basic drinking water (23%), sanitation (22%), and hygiene (33%) services (1). This deficit in WASH services compromises students’ learning environment (2) and is directly linked to preventable illnesses like diarrhea (3) and respiratory infections (4). Beyond the immediate impacts of illness, early childhood diarrhea and respiratory infection are associated with impaired school performance (5) and increased absenteeism (6), leading to downstream impacts on educational attainment (5, 7, 8) and future earnings (9, 10). Consequently, providing WinS remains a high priority for achieving global health and educational equity goals.

The primary goal of WinS investments is to support students’ learning, health, and dignity (2). However, realizing these benefits requires not only that school WASH facilities are present, but that students use them correctly and consistently. For instance, using the school toilet instead of defecating in the open, practicing consistent handwashing with soap at critical times, and choosing safe drinking water sources are key WASH behaviors with a robust evidence base linking them to significant disease reduction (4, 11–14). As a result, studies may measure behavior as an intermediate outcome, or process indicator, along the causal pathway from intervention to health (15). Behavior can also serve as a strategic alternative primary outcome since detecting child health impacts often requires large sample sizes and long measurement periods (16), and because health status related to WinS interventions can be confounded by external factors such as seasonal illness, weather (e.g., rainfall, heat), and a student’s home environment (17–20).

While these advantages make behavior an important metric, collecting high-quality behavioral data involves methodological trade-offs. Unannounced direct observation captures action in real-time and is often regarded as the most objective measure of behavior. However, direct observation is resource-intensive, requiring investments of funding, staffing, and time. Furthermore, direct observation is subject to reactivity, such as the Hawthorne effect, where individuals may alter their actions when they know they are being monitored (21). Given these methodological and cost constraints, many WinS studies utilize reported metrics of behavior collected through surveys or interviews. Data are generated through students describing their own behavior (self-report), students describing the behavior of their peers (peer-report), and teachers or other school staff describing student behavior (teacher-report).

Methodological critiques of reported data, particularly self-reports, cite its vulnerability to recall error (22) and social desirability bias (SDB) (23). SDB is defined as “the tendency of research subjects to give socially desirable responses instead of choosing responses that are reflective of their true feelings” (24). These critiques extend well beyond the WASH sector. Studies in alcohol abuse (25), HIV risk behaviors (26), and other health-related topics (27) have documented bias in self-reported behavior data. Some literature suggests using a well-trained interviewer, or data collection instruments that do not require an interviewer, and/or incorporating a social desirability scale into surveys may minimize this bias (24). Other research in the field of questionnaire design argues that SDB may be less prevalent than often asserted (28, 29); however, this literature is primarily based on studies of adult respondents in high-income countries. We hypothesize that SDB may be significantly more prevalent among children, particularly in the school environment, where students are routinely evaluated and conditioned to learn and reproduce norms or “correct” answers.

The objective of this study was to assess the relative validity of unannounced observations, student self-reports, peer-reports, and teacher-reports for measuring students’ WASH behaviors in the school environment. We conducted a sub-analysis of a prospective, four-arm, cluster-randomized controlled trial (cRCT) that assessed the individual and combined impacts of WASH education and operation and maintenance (O&M) of WASH infrastructure on student and teacher knowledge, WASH facility conditions, and student behavior in India (15). When designing the parent trial, we planned to triangulate observed and reported behavior data to enhance the credibility of our findings. Divergent conclusions from reported and observed measures of student behavior, however, prompted us to undertake further analysis along with literature review to better understand the conflicting results.

## 2 Methods

### 2.1 Primary data

#### 2.1.1 Interventions and sample frame

Complete details about the parent study design, school selection and randomization, and participant recruitment were published previously (15). In brief, the parent study employed a factorial design to evaluate two interventions, individually and combined, across four treatment arms. The first intervention was a 12-session, play-based WASH curriculum taught by trained teachers during school hours. The curriculum focused on identifying safe drinking water, knowing critical times for handwashing, using a toilet, and practicing good personal hygiene. The second intervention was an O&M service designed to improve the cleanliness and functionality of school WASH infrastructure. A part-time cleaner was assigned to each school and given responsibility for cleaning the school’s water source, handwashing station, and toilets. The cleaner also restocked soap and water for handwashing, ensured water was available in toilets for anal cleansing, and reported any non-functional infrastructure or supply issues to school administrators.

We conducted the study in 200 government primary schools in one district of Uttar Pradesh (UP), India. To form our study sample, we used India’s national Unified District Information System for Education (UDISE) database to identify all public primary schools in this district. This resulted in a list of over 6,000 schools, which was narrowed to 1,746 schools after applying the study’s inclusion criteria (government schools – not private, schools which reported presence of an improved water source and gender segregated toilets on school premises). We focused this study on only government primary schools, not private schools, since our implementing partner, World Vision – India (WV-I), works exclusively with government-run schools and financing of WASH O&M differs greatly between government and private schools. Thus, our study population was government primary school (grades 1–5) students and teachers. Within this district, we selected 5 non-contiguous blocks with probability of selection proportional to the number of schools in each block. We then took a SRS of 49 schools in each of the five blocks to form a preliminary study sample (*n* = 245). To ensure comparable access to relevant WASH facilities at baseline, WV repaired or upgraded school WASH infrastructure so that each school had an on-premises water source, handwashing station, and gender-segregated toilet facility. These repairs were performed over 1 year prior to the start of the interventions in this trial. We visited these 245 schools to verify reported infrastructure and enrollment and excluded schools which, upon observation, lacked improved sanitation or water sources (*n* = 23), had low enrollment (*n* = 19), were too remote (*n* = 1), and that exceeded WV’s renovation budget (*n* = 13) for a sample of 189 schools. Eleven non-randomly selected schools were co-selected with local government to form a final sample of 200 schools. Sensitivity analyses of these 11 non-randomly selected schools were conducted in a prior analysis and identified no significant differences between these schools and randomly selected schools across any primary outcomes (15). We formed four clusters of 8–13 schools per block which grouped schools that were in close proximity, not separated by major geographical barriers like roads or rivers when possible, and not all in block centers nor in the most isolated areas of the block to protect against creating systematically different clusters. We used a random number generator to assign each cluster to one of four study arms: (1) WASH curriculum plus O&M (“Curriculum + O&M”), (2) WASH curriculum only (“Curriculum”), (3) O&M only (“O&M”), and (4) a Control group. Because our clusters varied in size, the final number of schools in each arm varied (*n* = 52 for Curriculum + O&M; *n* = 47 for Curriculum; *n* = 53 for O&M; and *n* = 48 for control). The visible nature of the interventions prevented us from blinding school staff or field staff to treatment assignments.

At baseline, we drew a gender-stratified sample of 20 grade 1 and 20 grade 4 students at each school. Each sampled student who was absent during subsequent rounds of data collection was replaced with another student of the same gender and grade to limit attrition effects in the study. However, students were allowed to rejoin the study if they were present during a later round of data collection. In total, data was collected from 6,630 students at baseline, 7,242 at midline, and 8,140 at endline. Within this cohort, 58% (*n* = 4,761) were surveyed across all three rounds, 39% (*n* = 3,182) were surveyed in two rounds, and 17% (*n* = 1,365) were surveyed just once (see Supplementary Figure S1 or reference (15) for a full PRISMA-style flow diagram of student sampling and attrition).

#### 2.1.2 Outcomes

The primary outcomes of this trial were rates of student toilet use and handwashing in the school environment. We measured these outcomes via unannounced observations, student self-reports, student peer-reports, and teacher-reports of student behavior. All indicators are briefly defined in Table 1; extended definitions and question text are provided in Supplementary Table S1. These indicators were originally designed for triangulation, not direct comparison. As such, the response alternatives are not directly comparable, and we do not compare magnitudes of change across these indicators. For example, we would not expect the observed proportion of students using the school toilet during a 2-h observation period (defined as count of observed toilet visits divided by school population) to change at the same rate as student’s self-reported defecation behavior. However, each of our primary outcomes measure a comparable behavior (toilet use for defecation, toilet use for urination, toilet use for any purpose; handwashing with soap) among a random sample of students which provides a valid signal on student behavior across our sample population. Thus, we would expect these indicators to align in terms of detecting positive or negative effects. We compare the direction of effect, as well as trends in each study arm over time, suggested by each indicator.

**Table 1:** Indicator definitions.

Indicators	Definition
Student Toilet Use	
Observed student toilet use for defecation or urination within a 2-h observation period	Count of students who were observed entering the school toilets during a 2-h observation period, divided by the daily estimated attendance of the school.
Self-reported student toilet use the last time they needed to defecate at school	Proportion of students surveyed who reported using the school toilet the last time they needed to defecate at school.
Peer-reports of “almost none/none” of their peers go outside to defecate (not time-bounded)	Proportion of students surveyed who reported “almost none/none” of their peers go outside when they need to defecate.
Teacher-reports of students “almost never/never” going outside to defecate (not time-bounded)	Proportion of teachers surveyed who reported students “almost never/never” go outside when they need to defecate.
Toilet Availability	
Toilets observed to be unlocked	A binary indicator taking the value of 1 if at least one of the boys’, girls’, and/or all-gender toilets that were observed (up to one each) was unlocked at the time of observation, and taking the value of 0 otherwise.
Student Handwashing (HW)	
Observed student HW with water / water and soap near the toilet facility	Count of students who were observed washing their hands with water or water and soap during a 2-h observation period, divided by the estimated number of students attending the school on the day of observation.
Self-reported use of soap at last instance of HW at school	Proportion of students surveyed who reported using soap the last time they washed their hands at school.
Soap Availability	
Soap for HW observed to be available to students	A binary indicator taking the value of 1 if soap was observed on school premises at the time of unannounced observation, and taking the value of 0 otherwise.
Teacher is able to produce a bar of soap upon request	A binary indicator taking the value of 1 if at least one teacher surveyed was able to produce a bar of soap from an easily accessible location, and taking the value of 0 otherwise.

We collected observational behavior data through unannounced 2-h observations of student toilet use and student handwashing during school hours. Additional details are provided under Data Collection. We used structured surveys to collect self-reported measures of student toilet use and handwashing with soap, as well as student reports on how often their peers defecate outside school premises rather than in the school toilets. We also collected teacher-reported data on their class’s toilet use using a structured survey, which we also used as an opportunity to verify if a stock of soap was present on school premises by asking each teacher to show the interviewer where soap was stored. We targeted students in first and fourth grades and their teachers for surveying, as these represented the upper and lower grades for which the WASH curriculum was designed. We directly measured toilet accessibility and soap availability for students through unannounced infrastructure observations.

Self-, peer-, and teacher-reports were framed around student defecation, whereas observed data on toilet use could not discriminate between urination and defecation. We collected and analyzed equivalent self-, peer-, and teacher-reported data on urination that are reported in Supplementary Figure S2 and Supplementary Table S2.

#### 2.1.3 Data collection

We launched both interventions in the Autumn of 2022, immediately after collecting baseline data. Approximately 6 months later we collected midline measurements, at a point when all key WASH concepts in the curriculum intervention were scheduled to have been introduced. We took endline measurements about 1 year after baseline, once schools had completed all 12 curriculum sessions (Supplementary Figure S3). The O&M service for school WASH facilities remained active until all endline data were collected.

In partnership with Oxford Policy Management - India (OPM), we completed a total of 6,630 student surveys at baseline, 7,243 at midline, and 8,140 at endline. We also conducted an average of 394 teacher surveys (2 teachers per school) at each round, and 101 cleaner surveys (1 per school) at endline. To avoid disturbing students’ normal behavior, we performed behavioral observations on a different day than student surveys. These observations were conducted near baseline, midline, and endline, and also every 3–4 weeks between rounds of surveying (*n* = 8 observations per school in total). Behavior observations were conducted for a period of 2 h near the school toilet facility to target handwashing after toilet use. Enumerators underwent rigorous training on how to position themselves discretely within the school and how to respond if questioned. Observations were performed early in the day or after the mid-day meal to avoid instances of group handwashing or toilet use at mealtimes, and instead to target individual hygiene choices. Observations were collected via paper and pencil tallies to reduce the chance of the observer being detected. The observation data were entered on a tablet using CSPro immediately after leaving the school premises.

In addition, we conducted short, unannounced infrastructure observations at baseline, midline, and endline, and also every 2–3 weeks between rounds of surveying (*n* = 11 observations per school). Several strategies were employed to mitigate reactivity, or the tendency for individuals to modify their behavior when being observed (30), among students and school staff. Observers collected data on soap availability as soon as they entered a school, before staff could restock it in response to their arrival. Additionally, a different enumerator visited each school at each round of observation. We held nightly debriefs with the observational data collection teams to troubleshoot issues and answer questions.

Additional details about formative work and the design of the O&M intervention (31) and study attrition (15) have been published previously.

#### 2.1.4 Statistical analysis

We conducted all analyses using an intention-to-treat approach and a difference-in-differences (DiD) framework. We modeled self-reported and teacher-reported outcomes with a generalized linear mixed effects model including a random effect for school ID and fixed effects for treatment condition, time, and block. Peer-reported outcomes were modeled with a cumulative link mixed model for ordinal variables including a random effect for school ID and fixed effects for treatment condition, time, and block. Lastly, we modeled behavior outcomes with generalized linear mixed models using Template Model Builder (glmmTMB) to handle overdispersion in the data. Models contained a random effect for cluster ID and a fixed effect for block. We accounted for the hierarchical nature of our data by including random effects in our models and by computing cluster-robust standard errors to produce more conservative *p*-values.

Some variables are reported as treatment-level averages rather than modeled data. Self-reported student handwashing with soap and self-reported student toilet use stratified by soap and toilet availability had uneven sample sizes across subsets that could have produced biased model estimates. Additionally, soap availability had insufficient variability to be modeled. Thus, we report findings for these variables as averages (percentages), versus modeled data which are reported in units of predicted probability. We performed the comparative analysis presented in this paper after unblinding treatment assignments. All analyses were carried out in R Version 2023.06.0 + 421.

### 2.2 Literature review

In addition to utilizing our own primary data in this sub-analysis, we leveraged Bick et al.’s (32) scoping review of health, education, and gender equity outcomes in WinS trials to analyze WASH-related behavior change interventions in the literature more broadly. In particular, we focused on associations between data collection methods and behavioral outcomes. We included articles from Bick et al. (32) which were identified as reporting at least one knowledge or behavior outcome. We also re-ran Bick et al.’s (32) search terms in the same six databases (Web of Science, Global Health, SCOPUS, Cochrane Library, MEDLINE, and Embase) to identify relevant articles published since the authors’ original search date (November 27th, 2023). Our search was bound strictly to the primary electronic database outputs; supplementary examinations of cited reference lists or citing references were not conducted, nor did we include additional studies or data based on contact with authors. The full lists of search terms for each database are provided in Supplementary Material SM2 and the PRISMA-S checklist is provided as Supplementary Material SM3. We performed our search on August 25th, 2025 and restricted the search to articles published since November 28th, 2023. De-duplication was performed in Zotero. Title, abstract, and full-text screening was conducted in Microsoft Excel using the inclusion criteria from Bick et al. (32). Two investigators were involved in screening and data extraction.

For each of the included articles, we extracted all behavioral outcomes and recorded how they were measured: reported data, direct observation, or with sensors. The WASH-related behaviors we analyzed included handwashing, toilet use, contact with safe/unsafe water sources, toothbrushing, face cleansing, and cleaning of the compound/school. Sensor measurements of behavior, such as sensors on soap dispensers, were included and classified as observational data as they provide an objective record of the behavior. Proxy measures of behavior, such as the concentration of fecal indicator bacteria in a hand rinse, were not included, nor were “technique” scores where students were prompted to wash their hands and tested on their knowledge of correct handwashing steps. Reported data were generally collected through in-person surveys or interviews with students, school staff, and parents. We summarized the effect of each intervention by quantifying the proportion of behavior outcomes within each study with a statistically significant positive effect reported by the authors. For example, a study which measured four behavior indicators via reported data and concluded two statistically significant positive findings would be summarized as 50% positive findings. We used this approach, rather than reporting the direction of effect of every behavior indicator measured across every study, to avoid over-weighting results from studies that measured a larger number of outcomes. In studies where behavior outcomes were measured by both reported and observed data, we summarized intervention effects within each measurement method. This analysis is not a formal meta-analysis, but rather a characterization of intervention effects to contextualize the primary data presented in this study. All extracted data is reported in Supplementary Material SM1.

### 2.3 Ethics statement

We secured ethical clearance for this investigation through Stanford University (California, United States, protocol 69,912) and Sigma Institutional Review Board (IRB) (India, protocol 10,046/IRB/22-23). Prior to engaging with the participating schools, we sought and received permission from officials within the UP Department of Education. Our team acquired written consent from all headmasters, teachers, and the parents/guardians of participating students at the project’s outset. For parents who could not read or write, teachers explained the consent forms orally and collected a fingerprint in lieu of a signature. Additionally, each student provided oral assent before participating in any survey activities. Following the completion of all endline data collection, we delivered the curricular intervention to control schools. Collected data were stored in OPM’s secure Dropbox and a de-identified data set was transferred to Stanford’s secure Google Drive.

## 3 Results

### 3.1 Primary data

We report magnitudes of change in this section to facilitate comparisons across treatment arms within an indicator. However, we reiterate that only the direction of effect is comparable across behavior indicators, not the magnitude.

#### 3.1.1 Reported data

**Figure 1:**
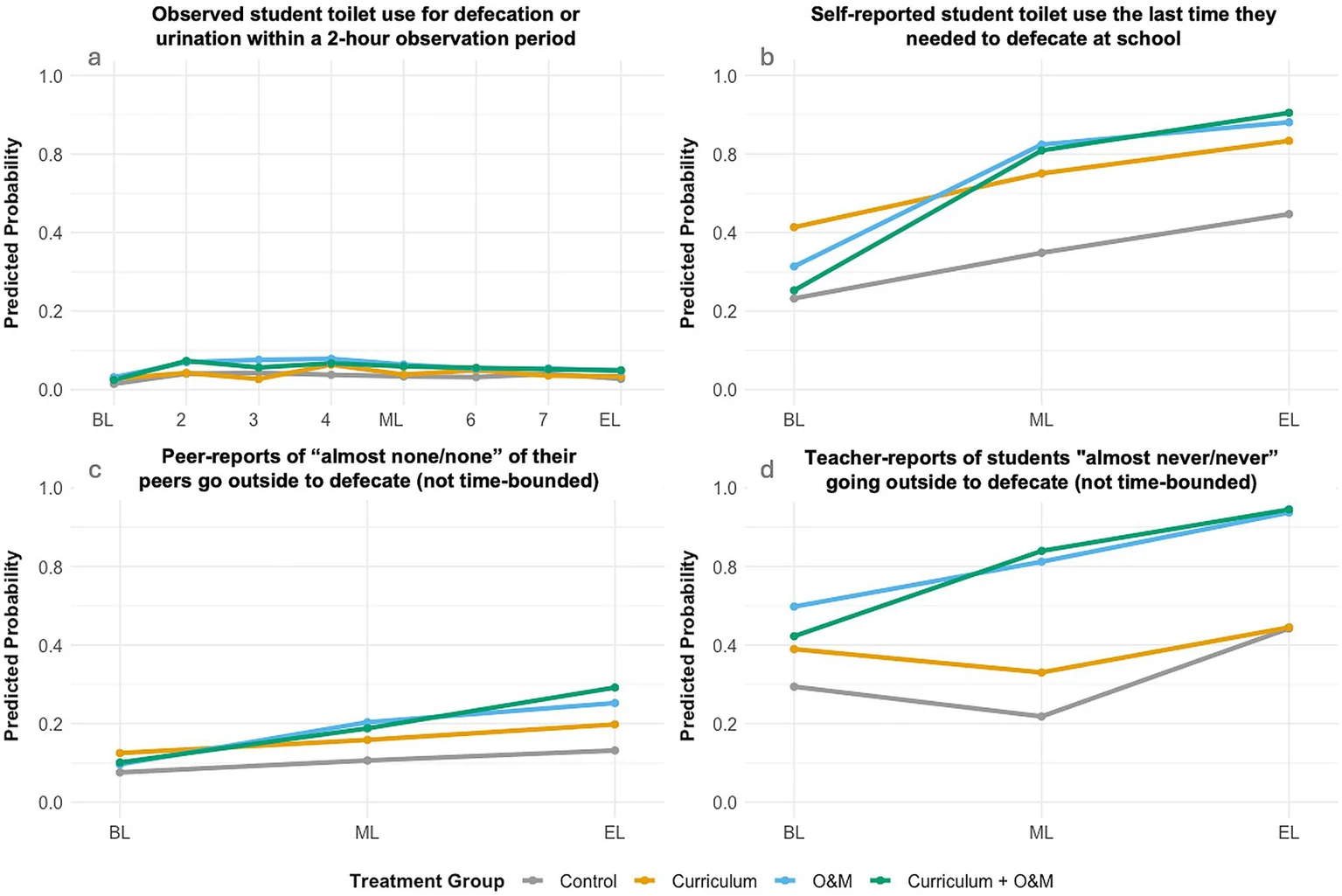
Line plots of **(a)** observed student toilet use for defecation or urination within a 2-h observation period, **(b)** self-reported student toilet use the last time they needed to defecate at school, **(c)** peer-reports of “almost none/none” of their peers go outside to defecate (not time-bounded), and **(d)** teacher-reports of students “almost never/never” going outside to defecate (not time-bounded). Model results are reported as predicted probability. Specifically, results represent **(a)** predicted probability of a student using the school toilet to defecate or urinate, **(b)** predicted probability of a student reporting they used the school toilet last time they needed to defecate at school, **(c)** predicted probability of a student reporting “almost none/none” of their peers go outside to defecate, and **(d)** predicted probability of a teacher reporting their student “almost never/never” go outside to defecate at school. Observed data were analyzed at eight time points, whereas reported data were collected and analyzed at three points. “BL,” “ML,” and “EL” represent baseline, midline, and endline measurements, respectively. Time points 2–7 in panel **(a)** are observations collected between rounds of surveying. Observed behavior data were always collected on different days than survey data since surveying disturbed students’ normal behavior.

All three types of reported data (self-, peer-, and teacher-reported) indicated that statistically significant increases occurred in student toilet use in both the O&M and the Curriculum + O&M arms, but not in the Curriculum arm (Table 2). The Curriculum arm performed similarly to the Control across self-, peer-, and teacher-reported measures of student toilet use for defecation; both showed 7–27 percentage point (pp) increases in reported measures of toilet use at endline. The probability of students self-reporting that they used the school toilet the last time they needed to defecate increased by 19 pp. in the O&M arm (*p* < 0.001) and by 30 pp. in the Curriculum + O&M arm (*p* < 0.001), relative to the trend in the Control (Figure 1). Similarly, the probability of students reporting that “Almost none/None” of their peers (peer-report) went outside to defecate at school increased by 13 pp. (*p* < 0.001) and 17 pp. (*p* < 0.001) in the O&M and Curriculum + O&M arms, respectively, relative to the Control. Similarly, the probability of a teacher reporting that students in their class “Rarely or Never” defecate outside while at school increased by 11 pp. in the O&M arm (*p* < 0.001) and 22 pp. in the Curriculum + O&M arm (*p* < 0.001), relative to the Control. Across all reported behavior outcomes, the Curriculum + O&M arm exhibited effect sizes 35–91% higher than those in the O&M arm, although the study was not designed to compare these arms with hypothesis testing. Analysis of self-, peer-, and teacher-reported data on urination led to identical conclusions (see Supplementary Figure S2 and Supplementary Table S2).

**Table 2:** Summary of results for observed, self-reported, peer-reported, and teacher-reported toilet use models in units of predicted probability (0–1, continuous).

Outcome	Control		Curriculum			O&M			Curriculum + O&M		
	Baseline probability (95% CI)	Average change (pp)	Baseline probability (95% CI)	DiD change (pp)	*p*-value	Baseline probability (95% CI)	DiD change (pp)	*p*-value	Baseline probability (95% CI)	DiD change (pp)	*p*-value
Observed	0.02 (0.01, 0.04)	(+) 0.027	0.04 (0.02, 0.07)	(−) 0.010	0.45	0.04 (0.02, 0.07)	(+) 0.013	0.055	0.03 (0.01, 0.07)	(+) 0.016	0.065
Self-reported	0.29 (0.19, 0.41)	(+) 0.27	0.52 (0.39, 0.65)	(+) 0.01	0.27	0.39 (0.28, 0.52)	(+) 0.19	*p* < 0.001*	0.32 (0.21. 0.44)	(+) 0.30	<0.001*
Peer-reported	0.09 (0.07, 0.13)	(+) 0.07	0.16 (0.10, 0.24)	(+) 0.02	0.64	0.12 (0.07, 0.19)	(+) 0.13	*p* < 0.001*	0.13 (0.08, 0.20)	(+) 0.17	<0.001*
Teacher-reported	0.37 (0.21, 0.56)	(+) 0.19	0.49 (0.30, 0.68)	(−) 0.12	0.40	0.62 (0.44, 0.78)	(+) 0.11	0.04*	0.53 (0.35, 0.70)	(+) 0.22	0.003*

DiD change refers to the percentage point (pp) change calculated from difference-in-differences calculations which adjust for the background trend in the Control. Average change refers to the average pp. change across the entire study period among all schools in each treatment arm. For observed data, the average is calculated from the average of all post-baseline time points. For observed outcomes which exhibited linear trends, the average change was calculated from only endline values (endline minus baseline).

^*^Denotes a significant *p*-value (*p* < 0.05); *p*-values are associated with DiD change in probability of toilet use.

The unit for all point estimates and averages is predicted probability.

#### 3.1.2 Observed data

Observed data showed a small and non-significant increase in student toilet use (for either defecation or urination) in both the O&M and Curriculum + O&M arms. Adjusting for the trend in the Control, DiD estimates throughout the entire trial duration showed a maximum increase in student toilet use of 3 pp. within the O&M and Curriculum + O&M arms (Figure 1). The average increase across the trial was just 1.3 pp. (*p* = 0.055) and 1.6 pp. (*p* = 0.065) in the O&M and the Curriculum + O&M arms, respectively (Table 2). There was also no evidence of an enhanced effect on the rate of student toilet use in the Curriculum + O&M arm where both interventions were delivered concurrently. Compared to the Control, the Curriculum arm showed changes ranging from −3.6 pp. to 1.6 pp. across all time points, with an average change of −1.0 pp. (*p* = 0.448).

#### 3.1.3 Stress-testing reported outcomes

**Figure 2:**
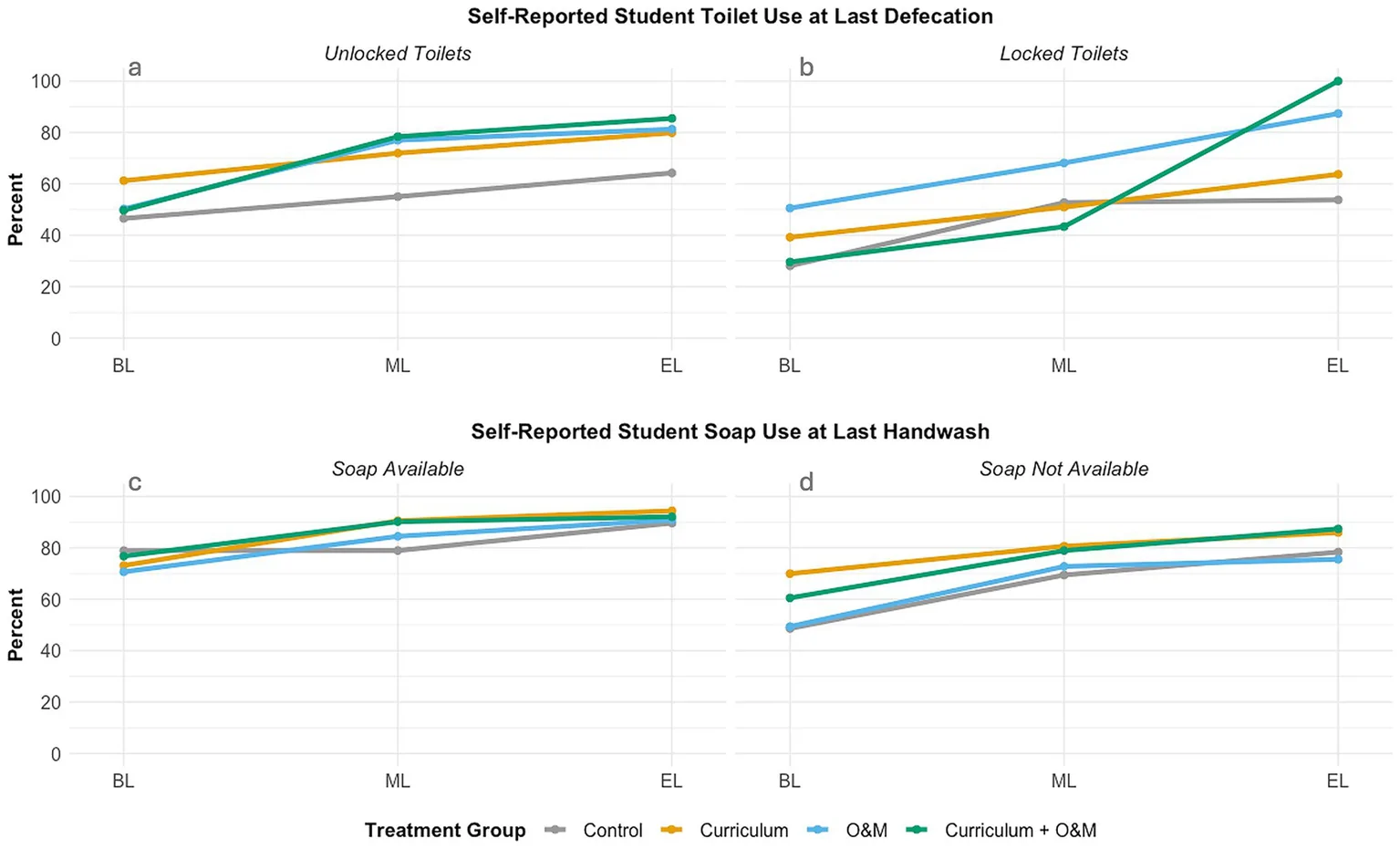
Line plots of student self-reported toilet use for defecation **(a,b)** and handwashing with soap **(c,d)** stratified by infrastructure observations. Panels **(a,b)** display student self-reports of whether or not they used the school toilet the last time they needed to defecate at school, stratified by whether toilets were observed to be unlocked **(a)** or locked **(b)** on the day of student surveying. Panels **(c,d)** display student self-reports of whether or not they used soap the last time they washed their hands at schools, stratified by whether soap was observed to be available (c) or not available (d) on the day of student surveying. All data is represented as percentages since group sizes were uneven and not well-suited for modeling.

Aiming to better understand the discrepancy between observed and reported data, we stratified students’ self-reported toilet use the last time they needed to defecate by whether or not the school toilet was observed to be unlocked at the time of their interview. We assume toilet availability to be constant across the course of a given school day since we only have one observation point just before the time of student surveys. However, our observational data supports the validity of this assumption since, across 3,188 h of behavior observations over the entire study period, we only recorded one instance of toilets being unlocked during the observation period. Students’ self-reported use of a toilet the last time they needed to defecate displayed little variation based on observed toilet availability (Figure 2). At baseline, an average of 38% (*n* = 1,101) of students in schools with locked toilets reported using the school toilet the last time they needed to defecate. At endline, on average 68% (*n* = 799) of students in schools with locked toilets reported using the school toilet the last time they needed to defecate, which was only 11 pp. fewer than the 79% (*n* = 7,331) of students who reported using the toilet in schools where it was unlocked (Supplementary Table S3). We performed two additional exploratory analyses to address the possibility that student self-reports referred to a defecation event that did not occur on the same day as their survey and the corresponding infrastructure observation. First, we expect that students urinate more often than they defecate at school, increasing the likelihood that a urination event happened on the same day as their survey. Analyzing self-reported location of last urination yielded similar findings to the defecation data (Supplementary Figure S4). Second, we stratified the self-reported defecation data by the time of day the survey was administered since we expect that fewer students might have defecated at school before morning surveys. Again, we found no meaningful differences between self-reported data collected in the morning versus the afternoon (Supplementary Table S4).

In a similar analysis, we stratified students’ self-reported use of soap for handwashing by whether or not soap was observed at the school that day (Figure 2). At baseline, an average of 56% (*n* = 4,314) of students reported soap use in schools where soap was not observed to be available that day (Supplementary Table S5). In comparison, an average of 75% (*n* = 1,570) of students reported soap use in schools that had soap available, a 19 pp. difference between strata. The gap in reported soap use between these strata narrowed to a 10 pp. difference by endline, with 82% (*n* = 5,274) of students in schools without soap reporting the use of soap compared to 92% (*n* = 2,188) in schools with soap.

To test the robustness of these findings, we broadened our definition of soap availability to include cases in which soap was not observed on school premises, but a teacher was able to produce a bar of soap from an easily accessible location at the end of their survey. Thus, schools classified as not having soap available were ones where soap was not observed nor could a teacher produce a bar from anywhere on school premises. This analysis resulted in similar findings as when soap availability was defined based only on observation of soap (Supplementary Figure S4). We also stratified self-reported soap use by the time of day a survey was administered since we expect that students interviewed in the morning would be less likely to have washed their hands at school on that day. Since we asked students what they used the last time they washed their hands at school, these students could have reported about a handwashing event that occurred on a different day than their interview and the corresponding infrastructure observation. We found no meaningful differences between self-reports collected in the morning versus in the afternoon (Supplementary Table S6).

#### 3.1.4 Other evidence of bias in primary data

We identified two additional instances of bias in our primary data. First, to compare self- versus peer-reported data on a behavior unrelated to the interventions in this trial, we asked students at endline about the frequency with which they and their classmates complete homework assigned at school. Students reported completing their own homework (70%) more frequently than their peers complete their homework (44%) (Supplementary Figure S5). In contrast to reported toilet use and soap use, neither self- nor peer-reported homework completion frequency varied across treatment conditions.

**Figure 3:**
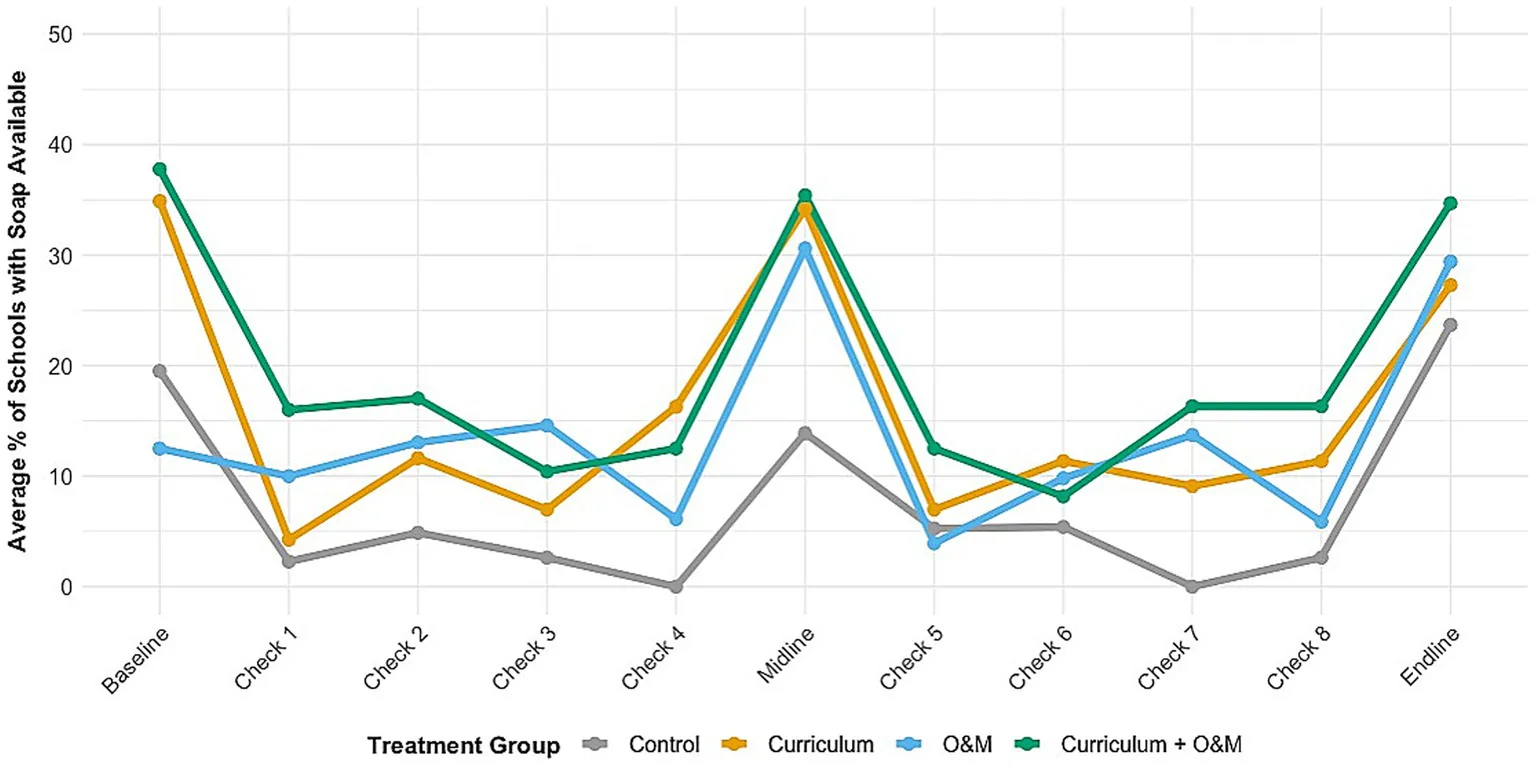
Line plot of average observed soap availability across treatment conditions at 11 time points. Baseline, midline, and endline observations were collected at scheduled visits, whereas Check 1–Check 8 were collected through unannounced visits. The *y*-axis displays the average percent of schools in each treatment condition that were observed to have soap available on school premises and the *x*-axis displays discrete observation times. Data is represented as percentages since the outcome was relatively rare and not well-suited for modeling.

We also found evidence of bias in observed data when schools were given advance notice of a field team’s visit. Specifically, we found systematic differences in the share of schools that had soap available for student handwashing between pre-scheduled visits and unannounced observations. During infrastructure observations that occurred on the same day as pre-scheduled student surveying (baseline, midline, and endline), soap was observed at 28% of schools on average (Figure 3). In contrast, during the 8 unannounced infrastructure observations conducted between rounds of student surveying only 9% of schools had soap available on average (Supplementary Table S7).

### 3.2 Comparative analysis of school-based WASH literature

**Figure 4:**
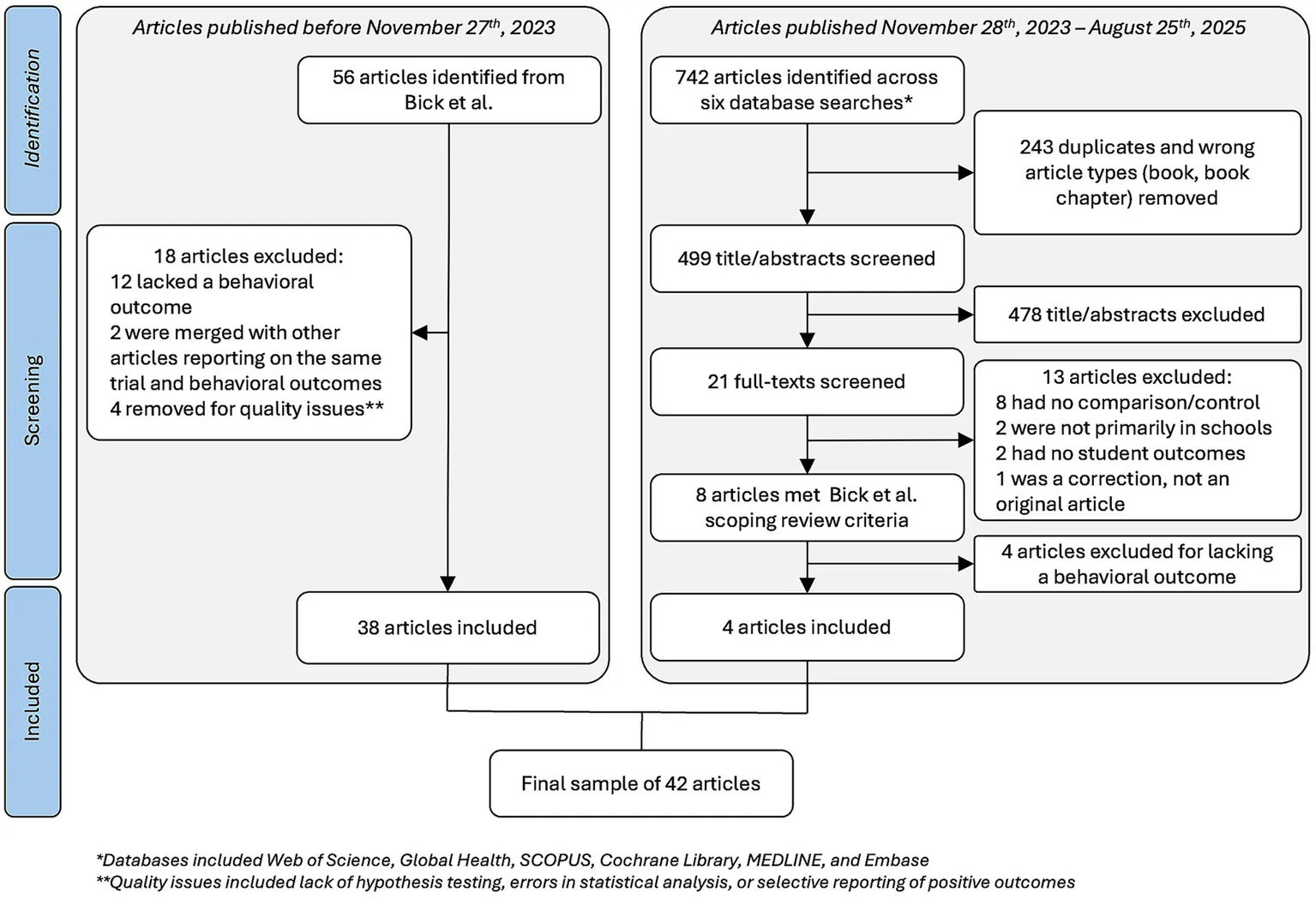
Flow chart showing the selection process for articles identified by the Bick et al. (32) scoping review and our updated search of articles published since their search in 2023.

To examine whether the discrepancies between observed and reported WASH behaviors in our primary data reflect a broader pattern in WinS research, we performed a scoping review of WinS literature focusing on behavioral outcomes. We identified 56 articles from the Bick et al. (32) scoping review that were classified as reporting knowledge or behavior outcomes. Our search of more recent literature across Web of Science and Global Health (*n* = 6), SCOPUS (*n* = 179), Cochrane Library (*n* = 135), MEDLINE (*n* = 162), and Embase (*n* = 302) returned 784 total articles. Of these, 285 duplicates were removed resulting in 499 unique articles. After screening, a total of 42 articles (33–74) were included in this review from the Bick et al. (32) scoping review and our updated search (Figure 4). Among these, 27 studies (64%) measured at least one behavioral outcome with reported data and 20 studies (47%) measured at least one behavioral outcome based on observations or sensors.

**Figure 5:**
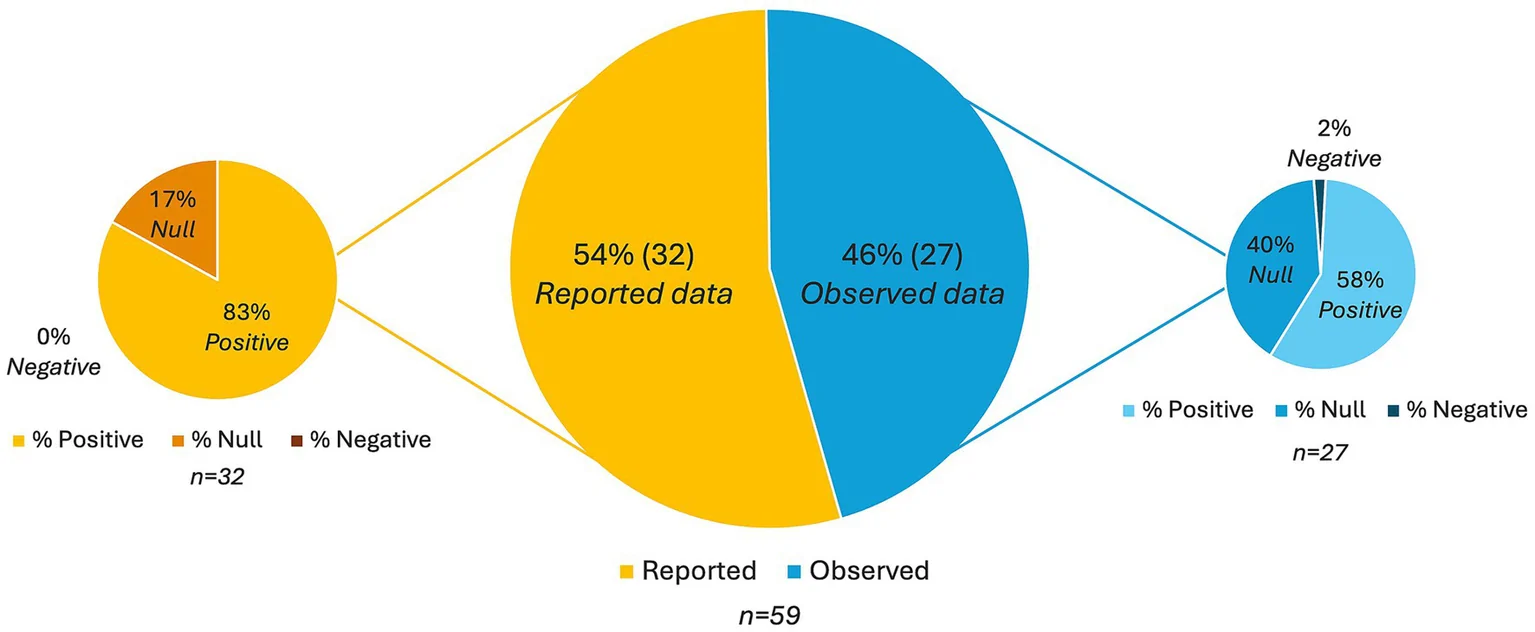
Results from a review of WinS literature that meets the Bick et al. (32) selection criteria and reports at least one behavioral outcome. The middle pie chart reports the proportion of intervention effects that were measured with reported outcomes versus observational data. The left pie chart presents the average proportion of intervention effects (within a study) from reported data that resulted in positive, null, and negative effects, and the right pie chart presents the same for intervention effects from observed data.

We identified a total of 59 intervention effects, of which 32 (54%) were measured with reported data and 27 (46%) were measured with observational data. The average proportion of positive (versus null or negative) intervention effects was considerably higher for outcomes measured with reported data (83%) as compared to outcomes measured through direct observation (58%) (Figure 5).

#### 3.2.1 Student behavior measured by direct observation or sensors

Next, we analyzed the subset of 20 WinS studies in our sample that included student behavior measurement via direct observation or sensors. These studies reported a total of 27 intervention effects. We also stratified the outcomes by intervention type: WASH education, infrastructure and supply provision, and ‘nudge’-based approaches (Table 3). We defined “nudges” as environmental or psychological cues intended to trigger a certain behavior. An expanded summary of the literature review results is provided in Supplementary Table S8.

**Table 3:** Summary of literature review findings among the subset of 20 articles that measured behavioral outcomes with observational data.

Study	Description of intervention(s)	Impacts on behavior outcomes
Education-based interventions: Curricula, educational campaigns, educational entertainment		
Appiah-Brempong et al. (36)	(1) Hand hygiene education (2) No intervention	Handwashing with soap (HWWS) after toileting increased; there were also smaller, but still statistically significant, increases in HWWS before meals
Chard and Freeman (37)	(1) Constructed school water supply, sanitation facilities, HW facilities, drinking water filters, and behavior change promotion (2) No intervention	No difference in HWWS after exiting the toilet
Duijster et al. (38)	(1) “FIT for school” school-based WASH education program (2) No intervention *All schools were provided soap for handwashing for measurement purposes	No difference in HWWS across arms
Graves et al. (39)	(1) Educational poster contests themed “HW with Soap” after installation of HW stations with treated water and soap (2) Installation of HW stations with treated water and soap	No difference in HW after toilet use across arms
Grover et al. (33)	(1) Simultaneous HW infrastructure and nudge* construction (2) Sequential HW then nudge* construction (3) Simultaneous infrastructure construction and high-intensity hygiene education (4) Sequential infrastructure construction and high-intensity hygiene education *Nudges included paved paths with painted shoeprints and arrows connecting latrines to handwashing stations, painted handprints on handwashing station with dedicated soap location	The high-intensity hygiene education intervention significantly increased HWWS after toileting at 5 months post-intervention. Simultaneous delivery significantly outperformed sequential delivery
Hussam et al. (40)	(1) Low-touch “edutainment” campaign with 15 min of weekly content, installation of a wall-mounted soap dispenser with embedded time-stamped sensor (2) Installation of a wall-mounted soap dispenser with embedded time-stamped sensor	HW at school increased while HW at home decreased in the intervention arm
Jetha et al. (41)	(1) 6-week HW campaign targeting social and emotional motivators of HW (2) No intervention	Small increases in the rate of HWWS and HW with at least water after toileting in the intervention arm
Lewis et al. (42)	(1) Unilever’s “School of 5” HW campaign (2) No intervention	No difference in HWWS before eating, HWWS after defecation, soap use while bathing, soap use at “other handwash times” among students or mothers across treatment arms
Patel et al. (43)	(1) Hygiene curriculum and installation HW and drinking water stations (2) No intervention	No difference in the proportion of students obtaining drinking water from school storage container
Sangalang et al. (44)	(1) High-intensity health education (*n* = 4 schools) (2) Medium-intensity health education (*n* = 7 schools) (3) Low-intensity health education (*n* = 2 schools) (4) No intervention (*n* = 2 schools) *All health education arms also received WASH policy workshops for teachers, hygiene supplies, and WASH facilities repairs	HW after toilet/urinal use increased among schools that received medium-intensity and high-intensity health education; no difference in HW after toilet/urinal use among schools that received low-intensity health education
Yuan et al. (45)	(1) Educational program using videos and comic books (2) No intervention	Use of safe water sources increased in the intervention arm
Infrastructure- and supply-based interventions: Infrastructure install or upgrades, soap provision, cleaning materials provisioning		
Bai et al. (46)	(1) Sequentially tested nature-background behind student faucets, then nature-background + spotlight (2) Sequentially tested wood-background, then wooden-background + auto-faucets (3) Sequentially tested spotlight then spotlight, nature-background, and auto-faucets (4) Sequentially tested auto-faucets then auto-faucets and spotlights (5) No intervention	All single interventions increased student HW, combined interventions had a larger impact on student HW rates; on average, student HW increased during summer months but was not significantly different during winter months
Bohnert et al. (47)	(1) Private sector service delivery (PSSD) of urine-diverting dry latrines with routine waste collection and maintenance (2) Government standard delivery of cistern-flush toilets or ventilated improved pit latrines	HWWS increased; no difference in toilet use and HW with water only with PSSD
Bulled et al. (48)	(1) Repair of HW taps near student toilets, construction of faculty toilets, one-time soap provision, one-time toilet cleaning, educational hand hygiene posters in toilets, WASH education program and classroom posters (2) WASH education program and classroom posters	Toilet use, HW after toilet use, and total HW increased in the combined infrastructure and education group; no difference in the education only group
Caruso et al. (49) and Saboori et al. (50)	(1) Latrine cleaning + HW (LC + HW): disbursement of latrine cleaning hardware and consumables, daily latrine cleanliness monitoring sheets for students, teacher training on latrine maintenance, provision of plastic bottles and powdered soap for HW (2) HW: provision of plastic bottles and powdered soap for HW (3) No intervention	HWWS after toilet use and HW with water only after toilet use increased in LC + HW and HW arms, no difference in latrine use across arms
Chard and Freeman (37)	(1) Constructed school water supply, sanitation facilities, HW facilities, drinking water filters, and behavior change promotion. Behavior change promotion entailed a daily group HW schedule, daily group compound cleaning schedule, and daily group toilet cleaning schedule posted at school. (2) No intervention	No difference in HWWS after exiting the toilet
Patel et al. (43)	(1) Hygiene curriculum and installation HW and drinking water stations (2) No intervention	No difference in the proportion of students obtaining drinking water from school storage container
Pickering et al. (51)	(1) Installation of wall-mounted hand sanitizer dispenser and teacher training (2) Installation of wall-mounted soap dispenser and teacher training (3) No intervention	Hand cleaning after toilet use and hand cleaning before lunch increased significantly in the sanitizer intervention schools. HWWS increased in the soap provision arm, but there was no difference in total hand cleaning
Wichaidit et al. (52)	(1) Install of the “Povu Poa” HW station with foaming soap and rinse water and behavior change promotion based on disgust and social norms (2) No intervention	HW with water only after toilet use and HWWS after toilet use improved in intervention schools
Nudge-based interventions: Cues, prompts, visual nudges		
Grover et al. (33)	(1) Simultaneous HW infrastructure and nudge* construction (2) Sequential HW infrastructure then nudge* construction *(3) Simultaneous infrastructure construction and high-intensity hygiene education* (4) Sequential infrastructure construction and high-intensity hygiene education *Nudges included paved paths with painted shoeprints and arrows connecting latrines to handwashing stations, painted handprints on handwashing station with dedicated soap location	The nudge intervention significantly increased HWWS after toileting at 5 months post-intervention. No significant differences were observed between simultaneous and sequential delivery of nudges
Huang et al. (34)	(1) School-based nudges: painted footpath from toilet to HW station, posters, eye sticker (2) No intervention	HWWS after toileting increased in “high WinS index schools” after installation of nudges; HWWS did not increase in “low-to-medium WinS index schools”
Naluonde et al. (35)	(1) Soap-on-a-roap installed (2) No intervention	Soap use increased in intervention schools; handwashing with water only did not change

Articles that appear in multiple intervention categories implemented multiple interventions within a single trial. Trials were only classified as having an infrastructure- or supply-based intervention if the effect of the infrastructure- or supply-based improvement was specifically tested (i.e., was not also implemented in the comparison arm).

##### 3.2.1.1 WASH education interventions

The largest sub-group (*n* = 11) included studies that used education-based interventions (*n* = 11). Sample sizes ranged from 2 to 98 schools per study arm (mean = 30, median = 16 schools), and measurement periods ranged from 2 weeks to 3 years (mean = 8 months, median = 4 months) (Supplementary Table S8). Five studies provided only educational inputs, and six provided educational interventions alongside some form of infrastructure or consumable support. Educational interventions took the form of traditional, classroom-based lessons at varied levels of intensity (*n* = 7), poster contests (*n* = 1), “edutainment” using videos and/or comic books (*n* = 2), or short campaigns targeting social and emotional motivators of behavior (*n* = 1). Studies in this sub-group reported on handwashing with soap (HWWS)/handwashing after toilet use (*n* = 7), HWWS before meals (*n* = 2), HWWS/handwashing at unspecified times (*n* = 3), handwashing at home (*n* = 1), soap use when bathing (*n* = 1), and use of safe water sources (*n* = 2). Nine studies measured some form of handwashing by observation or sensors and reported on 16 handwashing outcomes. Among these 16 outcomes, half (8/16) resulted in statistically significant increases in handwashing.

##### 3.2.1.2 Infrastructure and supply provision interventions

Eight studies tested infrastructure and supplies provision, although three were limited by small sample sizes of just 1 or 2 schools per study arm (Supplementary Table S8). Sample sizes ranged from 1 to 50 schools per treatment arm (median = 10, mean = 14 schools), and measurement periods ranged from 2 months to 3 years (median = 6 months, mean = 12 months). Interventions designed to improve student handwashing included installation of handwashing facilities or sanitizer dispensers (*n* = 4), soap provision (*n* = 3), repair of handwashing taps (*n* = 1), and improvements to handwashing station aesthetics (*n* = 1). Studies reported on HWWS/handwashing after toilet use (*n* = 5), HWWS/handwashing before eating (*n* = 1), HWWS/handwashing at unspecified times (*n* = 3), toilet use (*n* = 3), and use of safe water sources (*n* = 1). Seven studies measured some form of handwashing and reported 17 handwashing outcomes measured by observation or sensors. Among these outcomes, 65% (11/17) indicated statistically significant increases in handwashing, although measures and magnitudes of change varied. Interventions designed to increase student toilet use tested private sector service delivery (PSSD) of urine diverting toilets (*n* = 1), repair of school WASH facilities (*n* = 1), and toilet cleaning support (*n* = 1). PSSD and repair of WASH facilities resulted in increased toilet use, whereas toilet cleaning support did not.

##### 3.2.1.3 Nudge-based interventions

Three studies tested nudge-based behavior change interventions. Sample sizes in these studies ranged from five schools up to 66 schools per treatment condition, and measurement periods ranged from 3 to 5 months (Supplementary Table S8). These trials tested the effects of handwashing nudges in the presence of handwashing infrastructure. Nudges included paved footpaths, painted footprints and arrows, posters, stickers and painted handprints on handwashing stations, and hanging “soap on a rope.” One trial implemented nudges alongside high-intensity hygiene education. HWWS or soap use significantly increased in each of these trials, although HWWS only increased among “high WinS index schools” in one study (34).

## 4 Discussion

We presented a novel comparison of observed student toilet use for defecation or urination, self-reported student toilet use the last time they needed to defecate, peer-reported frequency of students using the school toilet to defecate (not time-bounded), and teacher-reported frequency of students using the school toilet to defecate (not time-bounded) in UP primary schools. These measurement methods led to fundamentally different conclusions about the effects of WASH education and facility O&M on student behavior. Had we relied solely on reported data, we would have concluded that the O&M intervention, both alone and combined with the WASH curriculum, resulted in significant increases in student toilet use. Further, we would have identified a synergistic effect of implementing O&M and education in tandem, confirming both of our core *a priori* hypotheses. In contrast, our observational behavior data revealed no evidence of a significant effect of O&M nor a magnified effect of the combined intervention. Stress-testing students’ self-reported toilet use and handwashing with soap against observations of toilet access and soap availability, respectively, brought the validity of self-reported data into question. Across all treatment conditions and both pre- and post-intervention, students frequently reported using toilets that were observed to be locked on the day of their interview and using soap when it was not observed to be readily available.

Our results are consistent with the well-documented phenomenon of social desirability bias (SDB) (23), but we observed variation in the magnitude of such bias both across time and with exposure to treatment. Over the course of the study, the share of students who reported engaging in socially desirable behaviors such as handwashing with soap and using the toilet for defecation gradually increased for all four study arms. Such gains were of course hypothesized in schools that received an intervention, but they were also observed in Control schools. This trend was most evident in the subset of Control schools without soap available, where students’ self-reported soap use increased 29 pp. on average from baseline to endline despite receiving no intervention and lacking soap.

Students in treatment arms also differentially reported desirable answers for behaviors that were related to the intervention that their school received. We hypothesize that the magnitude of bias is associated with how visible, disruptive, and consistently present a treatment is. While there is limited literature explicitly quantifying how intervention visibility may drive biased reporting, our hypothesis is grounded in prior evidence showing that perceptions of acceptable behavior (i.e., injunctive norms) play a role in over-reporting of handwashing (75). We documented a greater discrepancy between reported and observed behavior among students whose schools received the O&M intervention, where the daily presence of cleaners provided a constant reminder of the “desirable” behaviors being measured. The differential response bias reported in this study is particularly problematic for intervention evaluations because, if left undetected, it leads to erroneously reported impacts that are consistent with investigators’ *a priori* hypotheses.

It is also important to note that the manifestation of SDB may vary across institutional and environmental contexts. For example, prior public health literature has documented response bias in studies of alcohol abuse (25), HIV risk behaviors (26), and other health-related topics published in the Cumulative Index to Nursing and Allied Health Literature (CINAHL) (27). We hypothesize that the school environment may uniquely amplify this phenomenon, in part because students are conditioned to memorize and reproduce “correct” answers in their classes, and also because of the strong power dynamic between teachers and students. School-based studies of physical activity (76) interventions, food waste (77), and nutrition (78) have also identified that students over-reported target behaviors. Identifying the conditions under which SDB in behavioral interventions is heightened, along with strategies to mitigate it, remain important areas for future research.

Whereas observation was the more valid source of behavior data in this trial, observational data can also suffer from bias. For example, a much larger share of schools had soap present at handwashing stations on days when they knew the data collection team would visit for student surveying (baseline, midline and endline) as compared to unannounced spot checks when soap was rarely observed. However, we did not conduct student behavioral observations at any scheduled observations, only unannounced observations, meaning we cannot comment on whether student behavior results would have followed a similar pattern as soap availability. Nonetheless, this evidence underscores the importance of rigorous observational methods to capture an accurate reflection of student behavior.

Although we feel our primary data analyses have strong internal validity, these findings alone, which represent a single field site in UP, India, are insufficient to conclude that this is a pervasive issue in applied WinS research more generally. Stepping beyond the scope of our primary data, our comparative analysis of WinS literature mirrors the findings from our primary analysis, suggesting that concerns about the validity of reported data likely extend beyond our specific dataset and field site. Across the 42 studies (33–74) and 59 intervention effects we reviewed, reported data yielded positive findings considerably more often than when WASH-related behavior outcomes were measured through direct observation or sensors. If we only analyzed findings from reported data in the literature, we would conclude that schools are a prime environment for health behavior change using a variety of intervention approaches. Instead, observational data on student behavior in the literature resulted in null or negative effects nearly as often as positive effects, suggesting that the mechanisms for achieving and sustaining behavior change remain poorly understood. One study in this review measured student handwashing with soap via both reported and observed data and found that students were 70 percentage points more likely to self-report handwashing with soap than was recorded via observation (41). Complementary to our primary data, this suggests that even when an intervention produces positive effects, reliance on reported data likely leads to a highly inflated perception of program efficacy. Together, these findings suggest that current WinS policy and practice may be grounded in an evidence base that varies significantly depending on the measurement tools employed, and that reliance on reported data may result in investments in ineffective interventions.

Re-evaluating the literature based only on findings from observational data provides clearer guidance for practitioners and researchers. The strongest, albeit limited, evidence points to nudge-based interventions, which resulted in uniformly positive and significant impacts on handwashing behaviors (33–35). However, evidence is lacking for if, and what kinds of, nudges might produce similar improvements in toilet use in areas where open defecation is still common. Education-based approaches are the most studied type of intervention, yet results are mixed. While higher-intensity education often produced larger and statistically significant effects on student behavior compared to lower-intensity educational programs, particularly when implemented alongside supportive infrastructure and consumables (33, 44), the evidence base is constrained by the lack of factorial designs to isolate intervention effects. Finally, infrastructure and supply provision displayed mixed results on handwashing and lacked comparable trials for toilet use. While supportive infrastructure is necessary for sustained improvements in handwashing, the inconsistent impacts on HWWS and individual practices indicate that other unidentified conditions are required to mediate behavior change. The lack of replicated trials for toilet use prevents a synthesis of reliable interventions, highlighting a need for more evidence on the conditions under which infrastructure investments successfully improve student toilet use.

We present evidence that high-quality, longitudinal observations of behavior produce more valid results than reported data, but such observations are a resource-intensive form of data to collect. Not all research studies will have the funding, staffing, and time to measure behavior through direct observation, and the current WinS literature provides examples of lower-cost ways to obtain reliable data. Studies have leveraged various types of sensors and cameras to measure proxies for direct observation of handwashing or toilet use, such as measures of entries and exits of toilet doorways and handwashing stations (33, 46), soap use through time-stamped sensors in soap dispensers (40), and before-and-after soap weight (38). Other proxy measures of WASH behaviors like hand cleanliness and dental plaque (13, 38, 50, 51) also have potential as objective measures of behavior, assuming the study design provides sufficient sample size and controls for estimating a reliable average effect across treatments.

### 4.1 Strengths and limitations

Strengths of this study include a long study duration, rigorous factorial design and implementation of the interventions, the frequency of reported and observational data collection, and the relatively large number of schools, students, and teachers in our sample. This study is also strengthened by building on the inclusive search strategy developed by Bick et al. (32) to capture a wide variety of school-based WASH studies that examined student WASH-related behaviors.

This study also has several limitations. The reported and observational behavior measures in our primary data were not designed for direct comparison, but rather triangulation on the direction of effect. The inconsistent response alternatives used for these indicators precludes us from testing for significant differences across types of observed and reported data. It is also important to note that questions about peer-reported toilet use and teacher-reported student toilet use for defecation were not bounded to a specific time period in the way our observational and self-reported data were. Since we are only comparing the direction of effect and not the magnitude of these indicators, we do not believe this undermines the validity of our comparison. However, we recommend time-bounded indicators for future replications of this study to allow for magnitude comparisons. Additionally, our observational data could not discriminate between urination and defecation for ethical reasons, whereas reported data reflect urination and defecation behavior individually. However, our analyses of reported urination data and reported defecation data against observed data, respectively, produced identical conclusions. Another important limitation is that students’ self-reported toilet use and handwashing were framed around the “last time” they defecated or washed their hands at school. It is possible that the event they recalled to us happened on a different day than their survey, and thus of our infrastructure observations. However, our follow-up analyses indicated that such incongruencies are likely very limited in the data. Similarly, we assumed that toilet availability (locked vs. unlocked) was consistent throughout a given school day. We presented evidence from our behavior observations demonstrating that school staff unlocking toilets in the middle of the day was very rare in this context, but we do not expect this assumption to hold in other settings. Sensor-based measurements of toilet availability could address this limitation in future studies. Finally, we also lacked data to stress-test the validity of our observational data in the way we stress-tested reported data with infrastructure observations. Future research could use hard-to-detect, continuous monitoring techniques such as videography to assess the validity of high-quality observational data collection.

The scope of our study is limited to one district of Uttar Pradesh, India, and is not generalizable to other geographies. However, our findings are strengthened by evidence from studies conducted in Ghana, Laos, Philippines, Kenya, Bangladesh, China, South Africa, and Zambia that were captured in our literature review. By design, this analysis was limited to data collected in the school environment. Future studies that evaluate discrepancies in observational and reported data in other countries and institutional contexts, such as healthcare facilities and the home environment, would strengthen our understanding of the limitations of reported data in measuring behavior more broadly.

## 5 Conclusion

Given the evidence of bias in our primary behavior data and the consistent trend across the WinS literature, we conclude that self-, peer-, and teacher-reported data yield unreliable estimates of both the magnitude and direction of effect on student behavior. We therefore recommend against the use of reported behavior data as primary outcome measures for WinS programs. We also advise against researchers attempting to derive a correction factor from this or other trials’ data to “deflate” reported behavior data via post-hoc statistical adjustment. The limited number of outcomes compared in this trial is not a reliable basis for statistical adjustment, and we hypothesize that the magnitude of bias in reported behavior data likely varies across contexts. Finally, the substantial increase in our Control arm among reported measures highlights the essential role of a control arm. Pre-post measurement is insufficient to produce valid results, particularly in an environment where students may be rapidly learning and reporting perceived desirable answers or social norms (79).

Stratifying the WinS literature to include only observational behavior findings reduced the body of evidence on school-based behavior change by over half. Some studies that lacked observational behavior data reported metrics of student knowledge, satisfaction, perceived safety, and other subjective outcomes that are appropriate to collect through reported data, and their findings on these metrics still add value to the literature base. However, it is important to acknowledge the tremendous amount of research funding that has been allocated to assess intervention effectiveness with unreliable, reported primary behavior outcomes. We argue that the WinS literature would benefit from a smaller quantity of properly powered studies which measure pre-specified outcomes along clearly defined theories of change with more objective measures of primary and intermediate outcomes. This paper also contributes a concise summary of the existing literature on WinS-related behavior change measured with observational or sensor data to guide future research and practice.

Finally, a great deal of WinS data collection happens at the state- and country-level, often in partnership with large intergovernmental organizations like the United Nations and World Health Organization. The Joint Monitoring Program (JMP) defines basic and advanced indicators for WASH services in schools that focus on service and facility indicators (1), since observational or sensor-based measures of facility use are not yet feasible for global monitoring efforts. Additional research should focus on measurement of JMP’s basic and advanced WinS indicators as process or intermediate indicators along the causal pathway from an intervention to changes in student behavior and health. This evidence will build a literature base that connects specific service and facility indicators to downstream impacts on health, educational attainment, and wealth.

## Data Availability

The raw data supporting the conclusions of this article will be made available by the authors, without undue reservation.
