# Supplementary material for: Methodology for measuring behavior change dictates the perceived effectiveness of school-based WASH interventions
Source: Front Public Health. 2026 Aug 31;14:1833899. doi: 10.3389/fpubh.2026.1833899 (PMC13572640; doi:10.3389/fpubh.2026.1833899)
Supplement: Supplementary file 1 [file Supplementary_file_1.docx]

***Supplementary Material***

# 1 Supplementary Figures and Tables

## 1.1 Supplementary Figures

**Supplementary Figure S1.** Conceptual model of how a WASH curriculum and O&M support lead to WASH-related behavior change in the school environment. Reprinted with permission from Hornsby et al. (15).

**Supplementary Figure S2.** Line plots of (a) observed student toilet use for defecation or urination within a 2-hour observation period, (b) self-reported student toilet use the last time they needed to urinate at school, (c) peer-reports of “almost none/none” of their peers go outside to urinate (not time-bounded), and (d) teacher-reports of students "almost never/never” going outside to urinate (not time-bounded). Model results are reported as predicted probability. Specifically, results represent (a) predicted probability of a student using the school toilet to defecate or urinate, (b) predicted probability of a student reporting they used the school toilet last time they needed to urinate at school, (c) predicted probability of a student reporting “almost none/none” of their peers go outside to urinate, and (d) predicted probability of a teacher reporting their student “almost never/never” go outside to urinate at school. Observed data were analyzed at eight time points, whereas reported data were collected and analyzed at three points. “BL”, “ML”, and “EL” represent baseline, midline, and endline measurements, respectively. Time points 2-7 in panel (a) are observations collected between rounds of surveying. Observed behavior data were always collected on different days than survey data since surveying disturbed students’ normal behavior. Y-axes are in units of predicted probability.

**Supplementary Figure S3.** Study timeline showing modes and timing of data collection.

**Supplementary Figure S4.** Students’ self-reported urination behavior stratified by toilet availability (a, b) and students’ self-reported soap use when imposing a stricter definition of soap availability (c, d). Soap was defined as available if it was observed on the school premises during infrastructure observations or if any teacher was able to produce a bar of soap from a location easily accessible by teachers or students at the end of their survey.

**Supplementary Figure S5.** Students’ self- and peer-reported homework completion across treatment conditions. Students were asked how often they complete their homework when it has been assigned (“Almost none of the times/None” “Sometimes,” “Almost all/All the times”) and the percent of students who responded “Almost all/All the times” is graphed by treatment. Students were also asked how many of the students in their grade/class complete their homework when it is assigned (“Almost none/None,” “Some,” “Almost all/All”) and the percent of students who responded “Almost all/All” is graphed by treatment.

## 1.2 Supplementary Tables

**Supplementary Table S1.** Extended definitions and question text for all indicators.

| **Indicators** | | **Question Text** | **Definition** |
| --- | --- | --- | --- |
| Student Toilet Use | Observed student toilet use for defecation or urination within a 2-hour observation period |  | Count of students who were observed entering the school toilets during a 2-hour observation period, divided by the daily estimated attendance of the school. |
|  | Self-reported student toilet use the last time they needed to defecate at school | Think about the last time you needed to defecate/poop when you were at school. Where did you go to defecate? | Proportion of students surveyed who reported using the school toilet the last time they needed to defecate at school. |
|  | Peer-reports of “almost none/none” of their peers go outside to defecate (not time-bounded) | Thinking about the children in your grade/class, how many of them go outside when they need to defecate? Would you say it’s almost all/all, some, almost none/none? | Proportion of students surveyed who reported “almost none/none” of their peers go outside when they need to defecate. |
|  | Teacher-reports of students "almost never/never” going outside to defecate (not time-bounded) | We have heard that some students at schools in this area don't use the toilet for urinating and defecating at school. Here at your school, how often do children go outside when they need to defecate? | Proportion of teachers surveyed who reported students “almost never/never” go outside when they need to defecate. |
| Toilet Availability | Toilets observed to be unlocked | Look at the entrance to the boys’/girls’/non-gender segregated toilets. Is it/Are they unlocked? | A binary indicator taking the value of one if at least one of the boys’, girls’, and/or all-gender toilets that were observed (up to one each) was unlocked at the time of observation, and taking the value of zero otherwise. |
| Student hand washing | Observed student handwashing with water / soap and water near the toilet facility |  | Count of students who were observed washing their hands with water or water and soap during a 2-hour observation period, divided by the estimated number of students attending the school on the day of observation. |
|  | Self-reported use of soap at last instance of handwashing at school | What did you use to wash your hands that time? Did you use water, soap, soil or ash, other? | Proportion of students surveyed who reported using soap the last time they washed their hands at school. |
| Soap Availability | Soap for handwashing observed to be available to students | Do you see soap available at this time? | A binary indicator taking the value of one if soap was observed on school premises at the time of unannounced observation, and taking the value of zero otherwise. |
|  | Teacher is able to produce a bar of soap | Can you show us where soap is kept? | A binary indicator taking the value of one if at least one teacher surveyed was able to produce a bar of soap from an easily accessible location, and taking the value of 0 otherwise. |

**Supplementary Table S2.** Summary of results for observed, self-reported, peer-reported, and teacher-reported toilet use for urination models in units of predicted probability (0-1, continuous). DiD change refers to the percentage point (pp) change calculated from difference-in-differences calculations which adjust for the background trend in the Control. Average change refers to the average pp change across the entire study period among all schools in each treatment. For observed data, the average is calculated from the average of all post-baseline time points. For observed outcomes which exhibited linear trends, the average change was calculated from only endline values (endline minus baseline).

|  | **Control** | |  | **Curriculum** | | |  | **O&M** | | |  | **Curriculum + O&M** | | |
| --- | --- | --- | --- | --- | --- | --- | --- | --- | --- | --- | --- | --- | --- | --- |
| **Outcome** | **Baseline probability**  **(95% CI)** | **Average**  **change (pp)** |  | **Baseline probability**  **(95% CI)** | **DiD**  **change (pp)** | **p-value** |  | **Baseline probability**  **(95% CI)** | **DiD**  **change (pp)** | **p-value** |  | **Baseline probability**  **(95% CI)** | **DiD**  **change (pp)** | **p-value** |
| Observed | 0.02  (0.01, 0.04) | (+) 0.027 |  | 0.04  (0.02, 0.07) | (-) 0.010 | 0.45 |  | 0.04  (0.02, 0.07) | (+) 0.013 | 0.055 |  | 0.03  (0.01, 0.07) | (+) 0.016 | 0.065 |
| Self-reported | 0.35  (0.21, 0.51) | (+) 0.33 |  | 0.48  (0.32, 0.64) | (+) 0.00 | 0.20 |  | 0.43  (0.29, 0.59) | (+) 0.16 | p<0.001* |  | 0.44  (0.29. 0.60) | (+) 0.16 | <0.001* |
| Peer-reported | 0.12  (0.08, 0.17) | (+) 0.09 |  | 0.15  (0.09, 0.25) | (+) 0.03 | 0.54 |  | 0.15  (0.09, 0.24) | (+) 0.13 | p<0.001* |  | 0.15  (0.09, 0.24) | (+) 0.14 | <0.001* |
| Teacher-reported | 0.33  (0.21, 0.56) | (+) 0.06 |  | 0.39  (0.23, 0.58) | (-) 0.03 | 0.42 |  | 0.53  (0.35, 0.69) | (+) 0.27 | <0.001* |  | 0.41  (0.26, 0.58) | (+) 0.34 | <0.001* |
| **Denotes a significant p-value (p<0.05); p-values are associated with DiD change in probability of toilet use.*  *Note: The unit for all point estimates and averages is predicted probability.*  *Note: Difference-in-differences (DiD) change is the average growth adjusted for growth in the Control, reported as pp change.* | | | | | | | | | | | | | | |

**Supplementary Table S3.** Self-reported defecation outcomes stratified by observed toilet availability.

| **Students' Self-Reported Toilet Use at Last Defecation** | | | | | | |
| --- | --- | --- | --- | --- | --- | --- |
| **Treatment Group** |  | **Time** |  | **Locked Toilets*** |  | **Unlocked Toilets*** |
| Control |  | Baseline |  | 28% (n=185) |  | 47% (n=1323) |
|  |  | Midline |  | 53% (n=584) |  | 55% (n=1143) |
|  |  | Endline |  | 54% (n=290) |  | 64% (n=1572) |
| Curriculum |  | Baseline |  | 39% (n=209) |  | 61% (n=1352) |
|  |  | Midline |  | 51% (n=157) |  | 72% (n=1533) |
|  |  | Endline |  | 64% (n=273) |  | 80% (n=1627) |
| O&M |  | Baseline |  | 51% (n=356) |  | 50% (n=1361) |
|  |  | Midline |  | 68% (n=204) |  | 77% (n=1694) |
|  |  | Endline |  | 87% (n=190) |  | 81% (n=1951) |
| Curriculum + O&M |  | Baseline |  | 30% (n=351) |  | 50% (n=1447) |
|  |  | Midline |  | 43% (n=113) |  | 78% (n=1794) |
|  |  | Endline |  | 100% (n=46) |  | 86% (n=2181) |
| **Based on observations performed on the same day as student surveys* | | | | | | |

**Supplementary Table S4.** Students’ self-reported defecation behavior stratified by the time of day of the survey.

| **Proportion of Students who Self-Reported Using the School Toilet** | | | | | | | |
| --- | --- | --- | --- | --- | --- | --- | --- |
| **Treatment Group** |  | **Time** |  | **Early Survey*** |  |  | **Late Survey**** |
| Control |  | Baseline |  | 48% (n=597) |  |  | 42% (n=911) |
|  |  | Midline |  | 52% (n=603) |  |  | 56% (n=1124) |
|  |  | Endline |  | 62% (n=1196) |  |  | 64% (n=666) |
| Curriculum |  | Baseline |  | 57% (n=1017) |  |  | 61% (n=544) |
|  |  | Midline |  | 70% (n=971) |  |  | 70% (n=719) |
|  |  | Endline |  | 78% (n=1078) |  |  | 78% (n=822) |
| O&M |  | Baseline |  | 52% (n=605) |  |  | 50% (n=1112) |
|  |  | Midline |  | 76% (n=1068) |  |  | 76% (n=830) |
|  |  | Endline |  | 80% (n=1053) |  |  | 83% (n=1088) |
| Curriculum + O&M |  | Baseline |  | 48% (n=1209) |  |  | 40% (n=589) |
|  |  | Midline |  | 77% (n=1097) |  |  | 75% (n=810) |
|  |  | Endline |  | 86% (n=1137) |  |  | 85% (n=1090) |
| **Early surveys are defined as surveys performed before the mean survey time (10:39am)*  ***Late surveys are defined as surveys performed after the mean survey time (10:39am)* | | | | | | | |

**Supplementary Table S5.** Self-reported handwashing stratified by observed soap availability.

| **Students' Self-Reported Soap Use at Last Handwash** | | | | | | |
| --- | --- | --- | --- | --- | --- | --- |
| **Treatment Group** |  | **Time** |  | **Soap Not Available*** |  | **Soap Available*** |
| Control |  | Baseline |  | 49% (n=1022) |  | 79% (n=275) |
|  |  | Midline |  | 69% (n=1178) |  | 79% (n=171) |
|  |  | Endline |  | 78% (n=1197) |  | 90% (n=318) |
| Curriculum |  | Baseline |  | 70% (n=912) |  | 73% (n=536) |
|  |  | Midline |  | 81% (n=915) |  | 91% (n=476) |
|  |  | Endline |  | 86% (n=1311) |  | 94% (n=483) |
| O&M |  | Baseline |  | 49% (n=1377) |  | 71% (n=191) |
|  |  | Midline |  | 73% (n=1216) |  | 85% (n=535) |
|  |  | Endline |  | 76% (n=1435) |  | 91% (n=627) |
| Curriculum + O&M |  | Baseline |  | 61% (n=1003) |  | 77% (n=568) |
|  |  | Midline |  | 79% (n=1116) |  | 90% (n=642) |
|  |  | Endline |  | 87% (n=1331) |  | 92% (n=760) |
| **Based on observations performed on the same day as student surveys* | | | | | | |

**Supplementary Table S6.** Students’ self-reported soap use stratified by the time of day of the survey.

| **Proportion of Students who Self-Reported Using Soap at Last Handwash** | | | | | | |
| --- | --- | --- | --- | --- | --- | --- |
| **Treatment Group** |  | **Time** |  | **Early Survey*** |  | **Late Survey**** |
| Control |  | Baseline |  | 56% (n=599) |  | 53% (n=918) |
|  |  | Midline |  | 73% (n=607) |  | 67% (n=1125) |
|  |  | Endline |  | 80% (n=1196) |  | 81% (n=667) |
| Curriculum |  | Baseline |  | 71% (n=1019) |  | 68% (n=546) |
|  |  | Midline |  | 84% (n=971) |  | 77% (n=722) |
|  |  | Endline |  | 89% (n=1079) |  | 85% (n=822) |
| O&M |  | Baseline |  | 56% (n=605) |  | 50% (n=1113) |
|  |  | Midline |  | 79% (n=1071) |  | 72% (n=831) |
|  |  | Endline |  | 84% (n=1053) |  | 75% (n=1090) |
| Curriculum + O&M |  | Baseline |  | 66% (n=1206) |  | 62% (n=591) |
|  |  | Midline |  | 83% (n=1099) |  | 82% (n=810) |
|  |  | Endline |  | 89% (n=1138) |  | 87% (n=1090) |
| **Early surveys are defined as surveys performed before the mean survey time*  ***Late surveys are defined as surveys performed after the mean survey time* | | | | | | |

**Supplementary Table S7.** Average observed soap availability across both scheduled and unannounced observations of school infrastructure and consumables.

| **Average Observed Soap Availability** | | | | | | | | |
| --- | --- | --- | --- | --- | --- | --- | --- | --- |
| **Time** |  | **Control**  (n=48) |  | **Curriculum**  (n=47) |  | **O&M**  (n=52) |  | **Curriculum + O&M**  (n=53) |
| Baseline* |  | 19.5% |  | 34.9% |  | 12.5% |  | 37.8% |
| Check 1 |  | 2.3% |  | 4.3% |  | 10.0% |  | 16.0% |
| Check 2 |  | 4.9% |  | 11.6% |  | 13.0% |  | 17.0% |
| Check 3 |  | 2.6% |  | 7.0% |  | 14.6% |  | 10.4% |
| Check 4 |  | 0.0% |  | 16.3% |  | 6.1% |  | 12.5% |
| Midline* |  | 13.9% |  | 34.2% |  | 30.6% |  | 35.4% |
| Check 5 |  | 5.3% |  | 7.0% |  | 3.9% |  | 12.5% |
| Check 6 |  | 5.4% |  | 11.4% |  | 9.8% |  | 8.2% |
| Check 7 |  | 0.0% |  | 9.1% |  | 13.7% |  | 16.3% |
| Check 8 |  | 2.6% |  | 11.4% |  | 5.9% |  | 16.3% |
| Endline* |  | 23.7% |  | 27.3% |  | 29.4% |  | 34.7% |
| **Baseline, midline, and endline infrastructure observations were performed on the same day as student surveys* | | | | | | | | |

**Supplementary Table S8.** Expanded summary of literature review findings among the subset of articles that measured behavior outcomes with observational data.

| **Study** | **Sampling & Measurement** | **Description of Intervention(s)** | **Impacts on Behavior Outcomes** | **Other Related Results** |
| --- | --- | --- | --- | --- |
| **Education-based interventions: Curricula, educational campaigns, educational entertainment** | | | | |
| Appiah-Brempong et al., 2020 | 4 schools;  n=2 per treatment;  2-week measurement period | (1) Hand hygiene education  (2) No intervention | **Handwashing with soap (HWWS) after toileting increased**; there were also smaller, but still statistically significant, **increases in HWWS before meals** | Large improvements in reported *intention* to wash hands after toileting and before meals |
| Chard and Freeman, 2018 | 100 schools;  n=50 per treatment;  3-year measurement period | (1) Constructed school water supply, sanitation facilities, HW facilities, drinking water filters, and behavior change promotion  (2) No intervention | **No difference in HWWS after exiting the toilet** | No impact on pupil absence, enrollment, dropout, grade progression, diarrhea, respiratory infection, conjunctivitis, STH infection reported in 2019 paper |
| Duijster et al., 2020 | 20 schools;  n=10 per treatment;  4-month measurement period | (1) “FIT for school” school-based WASH education program  (2) No intervention  *All schools were provided soap for HW for measurement purposes | **No difference** in **HWWS** across arms | HWWS after toilet use measured through weighing soap and 2-way sensor measurements of toilet entryway |
| Graves et al., 2012 | 21 schools;  n=10 intervention schools;  4-month measurements period | (1) Educational poster contests themed “HW with Soap” after installation of HW stations with treated water and soap  (2) Installation of HW stations with treated water and soap | **No difference** in handwashing (**HW) after toilet use** across arms | The number of handwashing stations and proportion of teacher-supervised stations increased in the arm with poster contest; no difference across arms on soap availability or visibility of handwashing stations. |
| Grover et al., 2018 | Randomly selected 20 schools;  n=5 per treatment;  5-month measurement period  unannounced observations | *(1) Simultaneous HW infrastructure and nudge* construction*  *(2) Sequential HW then nudge* construction*  (3) Simultaneous infrastructure construction and high-intensity hygiene education  (4) Sequential infrastructure construction and high-intensity hygiene education  **Nudges included paved paths with painted shoeprints and arrows connecting latrines to handwashing stations, painted handprints on handwashing station with dedicated soap location* | The high-intensity hygiene education  intervention significantly **increased HWWS after toileting** at 5 months post-intervention. Simultaneous delivery significantly outperformed sequential delivery | Nudges and HE were found to be equally effective at improving HWWS after toileting. |
| Hussam et al., 2023 | 150 classes across 26 schools;  n=76 edutainment classes and n=74 control;  5-month measurement period;  unannounced observations | (1) Low-touch “edutainment” campaign with 15 minutes of weekly content, installation of a wall-mounted soap dispenser with embedded time-stamped sensor  (2) Installation of a wall-mounted soap dispenser with embedded time-stamped sensor | **HW at school increased** while **HW at home decreased** in the intervention arm | Aimed to test the potentially inverse relationship of increased handwashing at school with handwashing at home |
| Jetha et al., 2021 | 196 schools;  n=98 schools per treatment;  3-month measurement period | (1) 6-week HW campaign targeting social and emotional motivators of HW  (2) No intervention | **Small increases** in the rate of **HWWS** and **HW with at least water after toileting** in the intervention arm | The number of HW stations stocked with soap increased in the intervention arm. Limited impact on social motivators suggesting differences in soap availability may have driven behavior change. |
| Lewis et al., 2018 | 32 villages containing at least 1 school;  n=16 villages per treatment;  8-10 week measurement period | (1) Unilever’s “School of 5” HW campaign  (2) No intervention | **No difference** in **HWWS before eating**, **HWWS after defecation**, **soap use while bathing**, **soap use at “other handwash times”** among students or mothers across treatment arms |  |
| Patel et al., 2012 | 42 schools;  n=21 schools per treatment;  2-year measurement period | (1) Hygiene curriculum and installation HW and drinking water stations  (2) No intervention | **No difference** in the proportion of students **obtaining drinking water** from school storage container | Reported an improvement in students’ handwashing technique and respiratory illness, but no impact on diarrhea, from reported outcomes. |
| Sangalang et al., 2022 | 15 schools;  Number of schools per treatment varied;  8-month measurement period | (1) High-intensity health education (n=4 schools)  (2) Medium-intensity health education (n=7 schools)  (3) Low-intensity health education (n=2 schools)  (4) No intervention (n=2 schools)  *All health education arms also received WASH policy workshops for teachers, hygiene supplies, and WASH facilities repairs | **HW** **after toilet/urinal use increased** among schools that received medium-intensity and high-intensity health education; **no difference** in **HW after toilet/urinal use** among schools that received low-intensity health education | No difference across arms for health literacy, undernutrition prevalence, HAZ, or BAZ.  High-intensity HE reduced overnutrition and severe dehydration.  Medium-intensity HE reduced severe dehydration.  Low-intensity HE reduced stunting and overnutrition. |
| Yuan et al., 2000 | 50 primary schools;  n=25 schools per treatment;  ‘1-month measurement period;  unannounced observations | (1) Educational program using videos and comic books  (2) No intervention | **Use of safe water sources increased** in the intervention arm | Knowledge of schistosomiasis improved in the intervention arm. |
| **Infrastructure- and supply-based interventions: Infrastructure install or upgrades, soap provision, cleaning materials provisioning** | | | | |
| Bai et al., 2022 | 1 school serving roughly 5,000 students;  30-week measurement period;  unannounced observations | (1) Sequentially tested nature-background behind student faucets, then nature-background + spotlight  (2) Sequentially tested wood-background, then wooden-background + auto-faucets  (3) Sequentially tested spotlight then spotlight, nature-background, and auto-faucets  (4) Sequentially tested auto-faucets then auto-faucets and spotlights  (5) No intervention | All single interventions **increased** **student HW**, combined interventions had a **larger impact on student HW rates**; on average, **student HW increased during summer months** but was **not significantly different during winter months** |  |
| Bohnert et al., 2016 | Randomly selected 20 schools;  n=10 schools per treatment;  1-year measurement period;  unannounced observations | (1) Private sector service delivery (PSSD) of urine-diverting dry latrines with routine waste collection and maintenance  (2) Government standard delivery of cistern-flush toilets or ventilated improved pit latrines | **HWWS increased**; **no difference** in **toilet use** and **HW with water only** with PSSD | Maintenance and pathogen exposure were similar across treatments |
| Bulled et al., 2017 | 2 schools;  n=1 per treatment;  3-month measurement period | (1) Repair of HW taps near student toilets, construction of faculty toilets, one-time soap provision, one-time toilet cleaning, educational hand hygiene posters in toilets, WASH education program and classroom posters  (2) WASH education program and classroom posters | **Toilet use**, **HW after toilet use**, and **total HW increased** in the combined infrastructure and education group; **no difference** in the education only group | Small improvements in HW were observed in education-only group. |
| Caruso et al., 2014 & Saboori et al., 2013 | 60 schools;  n=20 per treatment;  5-month measurement period;  unannounced observations | (1) Latrine cleaning + HW (LC+HW): disbursement of latrine cleaning hardware and consumables, daily latrine cleanliness monitoring sheets for students, teacher training on latrine maintenance, provision of plastic bottles and powdered soap for HW  (2) HW: provision of plastic bottles and powdered soap for HW  (3) No intervention | **HWWS after toilet use** and **HW with water only after toilet use increased** in LC+HW and HW arms, **no difference in latrine use** across arms | Latrine cleanliness significantly improved in LC+HW arm, no difference in absenteeism across arms |
| Chard and Freeman, 2018 | Randomly selected 100 schools;  n=50 per treatment;  3-year measurement period | (1) Constructed school water supply, sanitation facilities, HW facilities, drinking water filters, and behavior change promotion. Behavior change promotion entailed a daily group HW schedule, daily group compound cleaning schedule, and daily group toilet cleaning schedule posted at school.  (2) No intervention | **No difference in HWWS after exiting the toilet** | No impact on pupil absence, enrollment, dropout, grade progression, diarrhea, respiratory infection, conjunctivitis, STH infection reported in 2019 paper |
| Patel et al., 2012 | 42 schools;  n=21 schools per treatment;  2-year measurement period | (1) Hygiene curriculum and installation HW and drinking water stations  (2) No intervention | **No difference** in the proportion of students **obtaining drinking water** from school storage container | Reported an improvement in students’ handwashing technique and respiratory illness, but no impact on diarrhea, from reported outcomes. |
| Pickering et al., 2013 | 6 schools;  n=2 per treatment;  2-month measurement period;  unannounced observations | (1) Installation of wall-mounted hand sanitizer dispenser and teacher training  (2) Installation of wall-mounted soap dispenser and teacher training  (3) No intervention | **Hand cleaning after toilet use** and **hand cleaning before lunch increased** significantly in the sanitizer intervention schools. **HWWS increased** in the soap provision arm, but there was **no difference** in **total hand cleaning** | Observed rhinorrhea reduced in sanitizer schools. |
| Wichaidit et al., 2019 | Randomly selected 30 schools;  n=10 schools in each batch for sequential delivery;  24-week measurement period | (1) Install of the “Povu Poa” HW station with foaming soap and rinse water and behavior change promotion based on disgust and social norms  (2) No intervention | **HW with water only after toilet use** and **HWWS after toilet use improved** in intervention schools |  |
| **Nudge-based interventions: Cues, prompts, visual nudges** | | | | |
| Grover et al., 2018 | Randomly selected 20 schools;  n=5 per treatment;  5-month measurement period;  unannounced observations | (1) Simultaneous HW infrastructure and nudge* construction  (2) Sequential HW infrastructure then nudge* construction  *(3) Simultaneous infrastructure construction and high-intensity hygiene education*  *(4) Sequential infrastructure construction and high-intensity hygiene education*  **Nudges included paved paths with painted shoeprints and arrows connecting latrines to handwashing stations, painted handprints on handwashing station with dedicated soap location* | The nudge intervention significantly **increased HWWS after toileting** at 5 months post-intervention. No significant differences were observed between simultaneous and sequential delivery of nudges | Nudges and HE were found to be equally effective at improving HWWS after toileting. |
| Huang et al., 2021 | 132 eligible schools;  n=66 per treatment;  4-month measurement period;  unannounced observations | (1) School-based nudges: painted footpath from toilet to HW station, posters, eye sticker  (2) No intervention | **HWWS after toileting increased** in “high WinS index schools” after installation of nudges; HWWS did not increase in “low-to-medium WinS index schools” | Access to functioning handwashing stations with soap also improved in intervention schools. |
| Naluonde et al., 2019 | 50 government schools;  n=25 per treatment;  3-month measurement period | (1) Soap-on-a-roap installed  (2) No intervention | **Soap use increased** in intervention schools; handwashing with water only did not change | Handwashing without soap improved in both intervention and control schools. |

## 1.3 Supplementary Text

**Supplementary Section S1. School selection details**

We drew a sample of 200 schools shortly before India's COVID-19 lockdown began. After it was lifted in July 2020, staff from World Vision India began visiting schools to make WASH upgrades prior to launching the study. They discovered that 10 schools' facilities needed repairs that would require substantial time and resources to complete. Once removed, World Vision India asked the local block education officers to identify 10 alternate schools that met our eligibility criteria and added them to the sample. These purposively selected schools included 2 from Block 1; 1 from Block 2; 6 from Block 3; 1 from Block 4; and 0 from Block 5. In addition, we discovered that one school in the sample had been added inadvertently during fieldwork (rather than being identified through the UDISE database). In total, 189 of the 200 schools ultimately enrolled in the study were included in our preliminary list of 245 candidates.
